# Cytoskeleton-mediated autophagy regulation in neuroimmune contexts: molecular mechanisms and functional perspectives

**DOI:** 10.3389/fimmu.2026.1732775

**Published:** 2026-04-21

**Authors:** Xingyu Cao, Haolin Zhang, Juan Wang

**Affiliations:** College of Chemistry and Life Science, Beijing University of Technology, Beijing, China

**Keywords:** actin filaments, autophagy, intermediate filaments, microtubules, septins

## Abstract

This review systematically summarizes the central roles and molecular mechanisms of the cytoskeletal system—including actin filaments, microtubules (MT), intermediate filaments, and the Septin family—in the regulation of autophagy. The cytoskeleton not only provides a structural framework and facilitates transport for the autophagic process but also acts as a dynamic signaling hub, participating in every stage from autophagosome formation and cargo recognition to targeted trafficking and autophagosome-lysosome fusion. Actin filaments regulate the initiation of autophagy through dynamic assembly, Arp2/3-mediated nucleation, and mechanosensing. Microtubules drive the transport and localization of autophagosomes by relying on “dynamic instability” and the “tubulin code”. Intermediate filaments—such as vimentin—and septins influence autophagy flux by maintaining organelle integrity, forming molecular scaffolds, and establishing diffusion barriers on membranes. This review further discusses the functional implications of this regulatory network in diverse physiological and pathological neuroimmune contexts, including neurodegeneration and aging. Finally, we highlight that targeting the cytoskeleton–autophagy interaction axis may offer novel therapeutic strategies for related diseases.

## 1 Introduction

The cytoskeleton is a dynamic network composed of protein filaments that extends throughout the cytoplasm, providing structural support for the cell. It plays a pivotal role in most forms of intracellular transport, thereby facilitating processes ranging from vesicle biogenesis to the trafficking of cargo throughout the cell (1). Actin filaments and microtubules (MT) are two fundamental components of the cytoskeleton, both of which undergo continuous and precisely regulated remodeling to enable dynamic spatial organization and rapid reorganization of the cytoskeletal network (2). Notably, actin polymerization is required for the biogenesis of autophagosomes derived from the endoplasmic reticulum membrane (3).

Autophagy is a cellular degradation pathway in which cytoplasmic cargo is delivered to lysosomes, playing a critical role in maintaining cellular homeostasis and responding to environmental stress (4). Upon exposure to stress conditions, autophagy is induced, leading to the formation of double-membraned autophagosomes within the cell. Intracellular materials destined for degradation are sequestered by autophagosomes, which subsequently fuse with lysosomes (in animals) or vacuoles (in yeast and plants) for degradation (5). The resulting macromolecules are then released back into the cytoplasm for cellular reuse (6). Autophagy can be categorized into macroautophagy, microautophagy, and chaperone-mediated autophagy, depending on the mode of cargo delivery (7). Additionally, autophagy can be classified as selective or non-selective, depending on substrate specificity (7, 8). Different types of autophagy vary in their physiological functions and mechanisms of cargo delivery to lysosomes (9). The autophagic process generally involves five key steps: initiation, nucleation, elongation, closure, and fusion (5). The discovery of autophagy-related genes (ATGs) has revealed the molecular mechanisms underlying autophagy (10). During autophagy, related proteins are sequentially recruited to the pre-autophagosomal site (PAS) to form autophagy-related protein complexes (11). The PAS serves as the initial site for autophagosome formation, with autophagy-related proteins being hierarchically assembled at the PAS (12).

**Figure 1:**
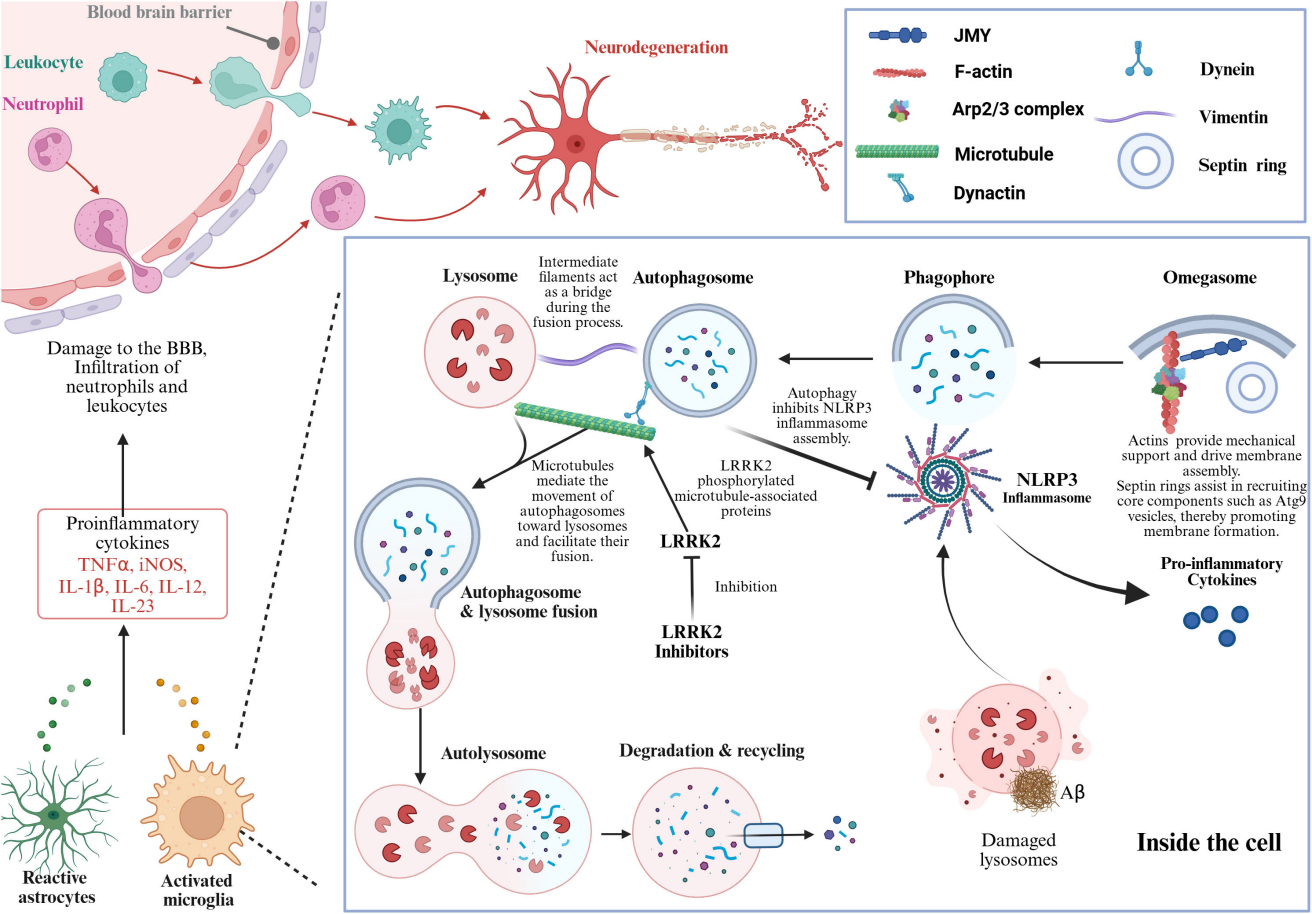
The core mechanism of the cytoskeleton-autophagy axis in regulating neuroinflammation and blood-brain barrier function. This figure illustrates the molecular network in which cytoskeletal components (actin filaments, microtubules, intermediate filaments, Septin rings, etc.) participate in the activation of neuroimmune cells, blood-brain barrier (BBB) damage and neurodegeneration by regulating the autophagy process under pathological conditions of the central nervous system. Reactive astrocytes and activated microglia release pro-inflammatory cytokines such as TNFα and IL-1β, which disrupt the integrity of the blood-brain barrier and lead to the infiltration of immune cells such as neutrophils and lymphocytes into the central nervous system. Within cells, microtubules act as transport tracks, mediating the movement of autophagosomes towards lysosomes and promoting their fusion; intermediate filaments play a “bridge” role in the process of autophagosome-lysosome fusion, ultimately forming autolysosomes to complete degradation and material recycling. Autophagy can inhibit the assembly of NLRP3 inflammasomes and reduce the release of pro-inflammatory factors; while LRRK2 protein phosphorylation inhibits autophagy, and its inhibitors can restore autophagic function. Created in BioRender.

The cytoskeleton serves as the fundamental structural platform regulating autophagy. Filamentous actin (F-actin) not only provides the mechanical support necessary for membrane expansion, but also participates in the formation and elongation of autophagic precursor structures, such as the phagophore (13). The polymerization and depolymerization state of F-actin directly influences the efficiency of autophagy initiation (13). Molecular motors, such as myosins, regulate the fusion of autophagosomes with lysosomes through their contractile and transport functions, with their activities being dependent on the integrity of the cytoskeletal network (3). Microtubules, functioning as “transport tracks”, mediate the directional movement of autophagosomes toward lysosomes and are therefore essential for the maturation and degradation of autophagosomes (14). The intermediate filament network, notably vimentin, provides mechanical support and spatial localization for organelles (15). In addition, this intermediate filament network acts as a bridge and stabilizing factor during the docking and fusion of autophagosomes with lysosomes, ensuring the proper progression of autophagic flux (16). The Septin family acts as an initiation scaffold for autophagy (17). At the PAS, septin rings serve as molecular scaffolds to facilitate the recruitment of core components of the autophagic machinery, including Atg9-containing vesicles, and promote membrane nucleation (**Figure 1**). Dynamic remodeling of the cytoskeleton—including polymerization, depolymerization, and rearrangement—is essential for each stage of autophagosome formation, maturation, transport, and degradation. Disruption of the cytoskeletal network impedes autophagosome transport, resulting in interrupted autophagic flux, accumulation of substrates, and ultimately exacerbated cellular stress and cell death.

Autophagy, as a crucial intracellular “clearance and recycling” system, forms a tightly interconnected bidirectional regulatory network with the neuroimmune system to maintain brain homeostasis, playing a central role in aging and neurodegenerative diseases (18). By eliminating aberrant proteins, damaged organelles, and senescent cells (19), autophagy negatively regulates neuroinflammation by suppressing the excessive activation of microglia and astrocytes (20, 21), and by blocking the activation of key pro-inflammatory signaling pathways such as the NLR Family Pyrin Domain Containing 3(NLRP3) inflammasome and NF-κB (22, 23), thereby maintaining immune balance. Conversely, the state of the neuroimmune system profoundly influences autophagic function (24). During neuroinflammatory processes, activated microglia and other immune cells release various pro-inflammatory cytokines (such as TNF-α and IL-1β), whose sustained secretion suppresses autophagic flux (25). Moreover, microglia can activate the mechanistic Target Of Rapamycin Complex 1 (mTORC1) pathway through receptors such as CCR5, further impairing autophagy and forming a vicious cycle (25).

The bidirectional regulation between autophagy and the neuroimmune system may also rely on the cytoskeleton as a functional bridge. The cytoskeleton not only provides the physical tracks required for the formation, elongation, and transport of autophagosomes (26), but its dynamic remodeling directly participates in regulating immune cell activation, migration, and phagocytic function (27). Disruption of the cytoskeletal system may hinder autophagic flux, exacerbate neuroinflammatory responses, and thereby establish a close pathological link among cytoskeletal abnormalities, autophagic dysfunction, and neuroimmune dysregulation. In the future, targeting the cytoskeleton as a central hub to coordinate autophagy and neuroimmune homeostasis may represent a promising therapeutic strategy to alleviate neuroinflammation and interrupt disease-associated vicious cycles.

## 2 Multiple regulatory roles of actin filaments in autophagy

Actin filaments, also known as microfilaments(MF) or F-actin, are polymers formed by the assembly of globular actin (G-actin) monomers (28). Actin is the most abundant protein in most eukaryotic cells and is among the most highly conserved proteins (29). Actin filaments exhibit remarkable dynamic behavior within the cell; their rapid assembly and disassembly enable them to quickly respond to intracellular and extracellular signals and participate in various essential cellular activities, such as the maintenance of cell morphology and intracellular transport.

Actin is a highly conserved globular protein with a molecular weight of approximately 42 kDa (30). There are three major actin isoforms in eukaryotic cells: α-actin, β-actin, and γ-actin (31). The assembly of F-actin is a tightly regulated, energy-dependent process that relies on ATP hydrolysis (32). This process generally involves three main stages (33): 1. Nucleation: This initial stage requires the assistance of nucleation proteins such as the Arp2/3 complex (34). 2. Elongation: ATP-bound actin monomers are rapidly added to the barbed (plus) end of pre-existing filaments. 3. Steady-state and ‘tread milling’: When the concentration of free monomers in solution reaches a critical level, the rate of addition at the barbed end equals the rate of dissociation at the pointed (minus) end, resulting in no net change in filament length. This dynamic behavior is termed “tread milling” (29).

During the biogenesis of autophagosomes, the membranes of these structures are typically derived from various organelle membranes, such as those of the endoplasmic reticulum, Golgi apparatus, and mitochondria, and this process is highly dependent on the driving force provided by actin filaments (35). Studies have demonstrated that actin is an essential component of autophagy: depolymerization of F-actin significantly inhibits the autophagic process (36). Furthermore, research has shown that actin filaments colocalize with crucial autophagy markers such as Microtubule-associated protein 1A/1B-Light Chain 3 (LC3), suggesting that actin may directly participate in the assembly and maturation of PAS (36, 37). In mechanotransduction-induced autophagy, actin filaments serve as the principal structural elements, with microtubules playing a supporting role (38). External mechanical compression can markedly increase the number of autophagosomes, whereas disruption of the actin network suppresses this effect (38). This indicates that an intact actin cytoskeleton is essential for sensing and transmitting mechanical signals to initiate autophagy. The structural integrity of actin filaments is closely associated with cellular elasticity; actin polymerization enhances cell rigidity, thereby increasing the sensitivity and efficiency of mechanotransduction, ultimately leading to more effective autophagy activation (38). Under simulated microgravity conditions, changes in F-actin tension alter autophagosome formation, transport, and fusion with lysosomes (39).

The nucleation factor Arp2/3 complex plays a crucial role in autophagy. Inhibition of the Arp2/3 complex or knockdown of Arp2 leads to a significant reduction in autophagosome-associated markers, such as LC3-II levels, as well as a marked decrease in the number of autophagosomes (40). Moreover, Arp2/3 inhibition induces the formation of elongated tubular structures that are positive for Double FYVE Containing Protein 1 (DFCP1) and LC3-II (markers of omegasomes), indicating that the Arp2/3 complex is also involved in the proper formation of omegasomes (40, 41). Additionally, the Arp2/3 complex can cooperate with its regulatory factors, such as junction-mediating and regulatory protein (JMY), also a p53 cofactor (42). The Arp2/3 complex promotes localized actin filament assembly during autophagy (43). During autophagy, LC3 recruits JMY to the phagophore and enhances actin-nucleating activity. Conversely, Tetratricopeptide Repeat Domain 5 (TTC5)/Tetratricopeptide repeat domain 5 (STRAP), a negative regulator of autophagy, binds to JMY and suppresses autophagy activation (43). The Arp2/3 complex, together with MYOsin 1D (Myo1D), mediates a physical clustering mechanism that fuses p62 nanodroplets into micrometer-scale condensates. This size-filtering process enables efficient LC3 labeling and subsequent degradation, thereby revealing the spatial and dimensional regulatory roles of actin filaments in controlling the fate of autophagic substrates (44). The Arp2/3 complex and its upstream activator WASP Homolog Associated with Actin, Membranes and Microtubules(WHAMM) are essential for lysosomal repair. Upon lysosomal membrane damage, they are rapidly recruited to the lysosomal surface, where they drive actin polymerization to repair the membrane and maintain lysosomal integrity. Their deficiency leads to the accumulation of numerous damaged and leaky lysosomes, which in turn completely blocks the degradation process of autophagolysosomes. As a result, autophagy stalls at the degradation stage, leading to the buildup of numerous undigested autophagolysosomes within the cell (45).

The Wiskott-Aldrich Syndrome Protein and SCAR Homolog(WASH) complex drives the recycling and transport of lysosomal hydrolytic enzymes (e.g., cathepsin D) by forming actin patches on endosomal membranes. Loss of its function traps these enzymes in late endocytic compartments, preventing their delivery to nascent autophagosomes. This results in a blockage of the autophagic degradation step, leading to “ineffective autophagy,” while causing the accumulation of undegraded substances — particularly lipids — within lysosomes (46). WASH protein, as a core component of the WASH-Arp2/3 complex, primarily regulates endosomal sorting and retrograde trafficking (47), This indirectly affects the localization of immune cell surface receptors (e.g., MHC class II) (48) This indirectly affects the localization of immune cell surface receptors (e.g., MHC class II) (49), thereby influencing the immune response.

Actin also plays a crucial role in neuroimmunity. Dynamic changes of actin regulate the assembly and localization of inflammasomes such as NLRP3, thereby affecting the activation of caspase-1 and the release of IL-1β and IL-18 (50). For example, F-actin depolymerization can promote the dissociation of NLRP3 from the cytoskeleton, enabling its association with Apoptosis-Associated Speck-like Protein Containing a CARD(ASC) to form inflammasome speckles (51). In macrophages, actin-dependent membrane ruffling/endocytosis also participates in regulating pathogen recognition (e.g., LPS recognition), indirectly triggering inflammasome activation (50). During antigen presentation, dendritic cells enhance their antigen-capturing capability through actin-mediated morphological polarization, such as the formation of dendritic extensions (52). When T cells form an immunological synapse with antigen-presenting cells (APCs), actin rearrangement generates a ring-like structure (cSMAC and pSMAC) (53), which is critical for TCR clustering, signal transduction, and the efficiency of antigen presentation (54). After antigen internalization, actin also drives the transport of endosomes to MHC II compartments, facilitating the assembly of peptide-MHC II complexes (55). Cytokine release is regulated by actin as well: the intracellular actin cytoskeleton provides transport tracks for cytokine vesicles (50)and participates in regulating the secretion process. Actin depolymerization can promote exocytosis and accelerate cytokine release (56). Additionally, the stabilization of GSDMD pores after inflammasome activation also relies on the actin cytoskeleton (57). In phagocytosis, the formation of phagocytic cups and the extension of pseudopods depend on actin polymerization, driven by complexes such as Arp2/3 (58). The maturation of phagosomes is also regulated by actin to facilitate fusion with lysosomes (59). During synapse pruning, microglia recognize and engulf synaptic debris via actin-driven pseudopod extension (60). In neurons, actin remodeling in the postsynaptic membrane affects the stability of dendritic spines, and its dysregulation may lead to excessive pruning, potentially contributing to conditions such as autism or epilepsy (61). In immune surveillance functions, NK cells and cytotoxic T lymphocytes (CTLs) rely on actin rearrangement to guide the directional transport of granzyme/perforin vesicles to the immunological synapse for targeted cell killing (62).

In summary, actin filaments are a highly dynamic core component of the cytoskeleton, and their finely tuned structure and function are fundamental for proper autophagic progression. From providing mechanical force for phagophore nucleation, facilitating selective autophagic substrate recognition and aggregation via the Arp2/3 complex, to integrating mechanical signals to regulate autophagic flux, actin filaments play irreplaceable and pivotal roles at multiple stages of autophagy.

## 3 The role of microtubules in autophagy

Microtubules are essential cellular components present in all eukaryotes, composed of heterodimers formed by the polymerization of α- and β-tubulin subunits (63). In addition, most eukaryotic cells contain multiple tubulin isoforms; for example, γ-tubulin is critical for microtubule nucleation, while δ-, ε-, and ζ-tubulin isoforms are typically found in cilia, flagella, and/or basal bodies, and are generally specific to organisms possessing these structures (64, 65). The dynamic instability of microtubules (polymerization and depolymerization) and the “tubulin code” (post-translational modifications such as acetylation and glutamylation) are key regulators of their functions (66).

During autophagy, microtubules play multifaceted regulatory roles. Dynamic microtubules are essential for the initiation of autophagy: under starvation conditions, the formation of autophagosomes requires dynamic microtubules, and either disruption or excessive stabilization of microtubules inhibits the accumulation of the autophagy-associated marker LC3-II (67). Early autophagy markers such as Unc-51 Like Autophagy Activating Kinase 1 (ULK1) and Beclin 1 are often associated with dynamic microtubules, providing a foundation for the assembly of PAS (67). Microtubules also regulate autophagic signaling through motor proteins. For example, under nutrient-rich conditions, kinesins position lysosomes at the cell periphery to maintain mTORC1 activity and suppress autophagy, whereas during starvation, lysosomes translocate toward the perinuclear region, resulting in mTORC1 inactivation and autophagy activation (68). After autophagosome formation, they must be transported along the microtubule network towards lysosomes, ultimately fusing with them to create autolysosomes for degradation of the cargo (14). Throughout this process, the integrity and dynamic remodeling of the microtubule network are crucial for efficient autophagosome transport, thereby ensuring proper clearance of cellular components and nutrient recycling via autophagy (14).

Furthermore, various post-translational modifications of tubulin, known as “tubulin code”, play important roles in regulating autophagosome transport and fusion. For example, acetylated microtubules facilitate the perinuclear transport of autophagosomes and their fusion with lysosomes, while detyrosinated microtubules influence lysosome positioning (69). In addition, polyglutamylated microtubules are closely associated with the transport of autophagosomes and fusion events between autophagosomes and lysosomes (70). Autophagosomes are transported bidirectionally along microtubules, relying on different motor proteins to move in opposite directions, ensuring their effective interaction with lysosomes (14). Microtubule-targeting agents and enzymes that modify tubulins play dual roles in cancer therapy by regulating autophagy: they can either inhibit autophagic flux to induce cell death or be exploited by cancer cells to promote their survival (68). Microtubule-targeting drugs and α-tubulin-modifying enzymes play regulatory roles in neuroinflammation and neurodegenerative diseases. Studies have shown that by stabilizing microtubules or modulating their post-translational modifications (such as acetylation and tyrosination), axonal transport can be improved, glial cell activation can be alleviated, and pathological protein deposition can be reduced. The core objective of microtubule-targeted therapeutic strategies in the management of neurological disorders lies in the restoration of neuronal homeostasis rather than the induction of cell death. Microtubule-stabilizing agents, typified by epothilone D, exhibit the capacity to permeate the blood-brain barrier and persistently accumulate within the brain parenchyma, exerting direct modulatory effects on the microtubule network in tauopathy models: these agents not only effectively augment microtubule density, ameliorate axonal transport deficits, and preserve structural integrity but also confer significant rescue of cognitive function, thereby embodying a therapeutic paradigm centered on functional compensation rather than cytotoxicity (71, 72).In this context, the post-translational modification (PTM) system of tubulin plays a pivotal role in fine-tuning microtubule physiology: for example, the compensatory upregulation of α-tubulin acetylation ratios observed in the brains of Alzheimer’s disease (AD) patients (73) and the precise spatiotemporal regulation of axon guidance mediated by polyglutamylation (74) collectively contribute to the maintenance of microtubule dynamic equilibrium and the promotion of neuronal viability. The restoration of such homeostasis subsequently exerts indirect regulatory effects on the neuroinflammatory milieu, fostering a coordinated “structure-function-immunity” neuroprotective axis. Accordingly, the therapeutic efficacy of these agents is highly contingent on the precise modulation of microtubule dynamic stability and PTM profiles, thus offering a promising interventional avenue for the disease-modifying treatment of AD and related tauopathies.

During meiosis, inhibition of PhosphoLipase D1 (PLD1) significantly enhances autophagic flux (75). This hyperactivated autophagy accelerates the degradation of key cytoskeletal regulatory proteins, such as Actin-Related Protein 2 (ACTR2), Phosphatidyldylinositol 4,5-bisphosphate (PtdIns (4, 5)P^2^), and phosphorylated-Cofilin 1(p-CFL1) (75). Loss of ACTR2 impairs vesicle transport and fusion, thereby affecting the dynamics of the F-actin network that drives spindle migration (75, 76); depletion of PtdIns (4, 5)P^2^ and p-CFL1 disrupts microtubule organizing center clustering and spindle assembly (75). Thus, PLD1 maintains cytoskeletal stability by suppressing excessive autophagy, ensuring proper spindle assembly, chromosome segregation, and cell polarity (75). During ciliogenesis, the microtubule-based transport system and autophagy receptors such as NudC Domain Containing 2 (NUDCD2) and NIMA-Related Kinase 9 (NEK9) cooperatively mediate the selective degradation of key proteins (e.g., Centriolar Protein 110kDa (CP110) and Myosin Heavy Chain 9 (MYH9)) to regulate cilium assembly (77, 78). Cilia themselves, as microtubule-based organelles, are capable of sensing mechanical signals and can activate autophagy via microtubule-dependent signaling pathways (such as Folliculin (FLCN)- AMP-activated Protein Kinase (AMPK)-mTOR), thus forming a positive feedback loop (79). Lysosomal inhibition leads to disruption of chromosomal and cytoskeletal structures, triggers both autophagy and apoptosis, and impairs porcine oocyte maturation and embryonic development (80). Autophagy can also regulate centrosome number (81), while centrosomes can modulate autophagy and lysosome biogenesis via the Polo-Like Kinase 4 (PLK4)- Transcription Factor EB (TFEB)/Transcription Factor Binding to IGHM Enhancer 3 (TFE3)signaling axis (82).

Microtubules mediate the intracellular transport of inflammasome components such as NLRP3 and ASC, ensuring their assembly at specific cytoplasmic locations (83). Microtubule depolymerizing agents (e.g., colchicine) can inhibit NLRP3 inflammasome activation and reduce IL-1β release (83). In dendritic cells, the intracellular transport of MHC class I molecules (from the endoplasmic reticulum to the Golgi apparatus and then to the plasma membrane) relies on microtubule-driven vesicle transport (84). When cytotoxic T lymphocytes (CTLs) form an immunological synapse with target cells, the microtubule organizing center (MTOC) polarizes toward the synapse, facilitating the directional transport of cytotoxic granules (85). The long-distance transport of cytokine vesicles (e.g., IL-6, IFN-γ) from the Golgi apparatus to the plasma membrane depends on kinesin/dynein movement along microtubules. Insufficient microtubule stability can lead to vesicle retention and reduced cytokine release (86). The directional polarization of the MTOC to the synaptic interface ensures targeted release of cytotoxic granules or exocytotic vesicles (87). After phagosome formation, microtubules mediate the transport of phagosomes toward the cell center, promoting their fusion with lysosomes (88). The chemotactic migration of neutrophils relies on microtubule-regulated establishment of cell polarity (89). During immunological synapse formation, microtubules also participate in phagosome maturation and transport to lysosomes (90). In neurons, microtubules mediate the transport of synapse-associated proteins, regulating the stability of dendritic spines (91). The migration of microglia depends on microtubule-driven organelle transport, supporting their pruning functions within the brain (92). The polarization of the MTOC in NK cells/CTLs relies on the microtubule cytoskeleton, ensuring precise release of cytotoxic granules toward target cells. The directional migration of macrophages depends on microtubule-regulated cell polarity, enabling effective tissue immune surveillance (93).

As a central component of the cytoskeleton, microtubules regulate autophagy through their dynamic instability, diverse isoforms, and the “tubulin code”—post-translational modifications such as acetylation and glutamylation. Microtubules participate in key stages of autophagy, including initiation, signal transduction, autophagosome transport, and autophagosome-lysosome fusion. Moreover, the interplay between microtubules and autophagy is extensively involved in essential cellular processes such as cell division, ciliogenesis, and centrosome function, collectively maintaining intracellular homeostasis. Notably, the precise regulation of microtubule dynamics and the tubulin code is equally critical in neuroimmune contexts, where it governs immune cell motility, synaptic plasticity, and organelle positioning—processes whose dysfunction is directly linked to autophagic failure.

## 4 The role of intermediate filaments in autophagy

Intermediate filaments, together with microfilaments and microtubules, constitute the cytoskeleton, providing cells with shape and structural integrity. By offering mechanical support, intermediate filaments protect cells from external forces and are involved in cell adhesion and tissue integrity (94). They form a widespread and complex network that acts as a molecular scaffold, organizing intracellular components and connecting the cell cortex to organelles within the cell (95). Early studies have demonstrated that the integrity of intermediate filaments is essential for the proper formation and distribution of autophagosomes (96). Treatment of rat hepatocytes with the protein phosphatase inhibitor okadaic acid (30 nM) leads to the disassembly of the keratin network into small spherical aggregates, accompanied by suppressed autophagy (97). Flavonoids such as naringin (100 μM) and protein kinase inhibitors such as KN-62 (10 μM) can preserve keratin structure and maintain autophagic activity (97). These findings suggest that the keratin cytoskeleton is rapidly regulated by phosphorylation and dephosphorylation and that keratin filaments may play a role in the autophagy process (97).

Subsequent studies have shown that autophagic vesicles are closely associated with vimentin (98, 99). Among the more than 70 intermediate filament proteins encoded in the human genome, Type III intermediate filament, vimentin, is the most widely distributed, spanning the cytoplasm and connecting with various organelles as a major cytoskeletal component (100). Vimentin dynamically regulates autophagy by modulating organelle localization, mTORC1 activity, and autophagosome–lysosome fusion (98, 101). Inhibition of vimentin with Withaferin A (WFA) causes vimentin to aggregate from a diffuse cytoplasmic distribution to a perinuclear region, concomitantly relocating autophagosomes (marked by LC3) and lysosomes (marked by Lysosomal-Associated Membrane Protein 1 (LAMP1)) to the perinuclear area (102). This abnormal distribution decreases spatial compatibility between autophagosomes and lysosomes in the cytoplasm, ultimately leading to intracellular accumulation of autophagosomes (33, 102). Vimentin also modulates mTORC1 activity and thus affects autophagy initiation. After WFA-induced vimentin aggregation, mTORC1 activity is suppressed, as evidenced by reduced phosphorylation of the downstream target rpS6, thereby relieving the inhibitory effect on autophagy, accelerating autophagosome formation in the early phase, and further exacerbating intracellular autophagosome accumulation (33). Using dual fluorescence labeling (GFP-RFP-LC3) and immunofluorescence assays, it has been demonstrated that vimentin dysfunction blocks autophagosome–lysosome fusion and impairs autophagic flux (33, 103). The regulation of autophagy by vimentin is “function-dependent”: the maintenance of its normal filamentous network structure is critical. Aggregation of vimentin disrupts autophagy, while simple vimentin deficiency has no significant effect on the autophagic process (33).

In macrophages, the intermediate filament protein vimentin can directly bind to and stabilize NLRP3, promoting its assembly and activation. Knockout of vimentin significantly inhibits NLRP3-mediated IL-1β release (104). In dendritic cells (DCs), vimentin is involved in anchoring MHC II molecules and co-stimulatory molecules, maintaining the distribution of membrane functional domains (105). In epithelial cells, the keratin cytoskeleton supports the structural integrity of endosomal compartments, facilitating transepithelial antigen presentation (106). Intermediate filaments regulate the nuclear translocation of signaling molecules such as NF-κB through anchoring. Vimentin also stabilizes the exocytotic machinery associated with TNF-α release, ensuring efficient cytokine secretion (107, 108). In macrophages, vimentin provides structural support for phagosomes, preventing their rupture (109). Upregulation of glial fibrillary acidic protein (GFAP) in microglia may affect their contact with synapses by maintaining cell morphology (110). Within neurons, the neurofilament network is crucial for maintaining axonal structural stability, and its dysfunction may indirectly impact synaptic stability (111). In cytotoxic T lymphocytes (CTLs), vimentin helps anchor the TCR signaling complex, enhancing signal transduction at the immune synapse (112). In natural killer (NK) cells, intermediate filaments can stabilize the storage structure of cytotoxic granules, supporting efficient targeted killing (113).

In the future, research into the relationship between intermediate filaments—such as keratins, vimentins, and others—and autophagy holds great promise. On one hand, it is necessary to further elucidate the precise regulatory mechanisms by which different intermediate filament proteins modulate distinct stages of autophagy. On the other hand, deciphering how intermediate filaments cooperate with microtubules and microfilaments to coordinate autophagy is of critical importance, particularly to clarify how these cytoskeletal components interact to influence autophagy in neuroimmune-related cells and under different physiological and pathological conditions.

## 5 The role of septins in autophagy

Septins are a conserved family of cytoskeletal proteins with GTPase activity, widely present in eukaryotes except for plants (114). Septins participate in numerous intracellular biological processes mainly by forming scaffolds or diffusion barriers (115). In budding yeast, seven septin subunits have been identified (Cdc3, Cdc10, Cdc11, Cdc12, Shs1, Spr3, and Spr28), while there are thirteen members in mammals (Septin1–Septin12 and Septin14) (116). These proteins display a high degree of sequence homology (117), revealing their evolutionary conservation and functional relevance. Different septin isoforms can assemble into heteromeric polymers, forming higher-order structures such as filaments or rings, which regulate a variety of cellular activities, including cell division, cell migration, apoptosis, microtubule regulation, membrane trafficking, vesicle transport, exocytosis, and autophagy (5, 7–9, 117). Aberrant expression or mutation of septins is linked to many diseases, including tumor metastasis, neurodegeneration, and immune dysfunction (118, 119).

In budding yeast, septins assemble early in the cell cycle at the site of the emerging bud, first forming a small patch that evolves into a ring structure before bud emergence (120). This ring subsequently matures into an hourglass-shaped collar in the cortex at the neck. The collar persists until just prior to cytokinesis, when it splits into two separate rings occupying opposite sides of the neck (120). Serving as a structural scaffold at the bud neck, septins recruit different components involved in various processes during distinct cell cycle stages, with many proteins asymmetrically associating with the septin ring (115). The scaffolding function of septin complexes operates in two main ways: first, by forming diffusion barriers (such as the septin ring at the bud neck), they prevent the movement of membrane-associated proteins between mother and daughter cells; second, as a polymeric scaffold, septins concentrate proteins required for critical cellular processes at specific subcellular sites (115, 121). Studies have shown that, upon autophagy induction, septins are not only localized at the bud neck but also found throughout the cytoplasm, dynamically shuttling among different organelles such as the Golgi apparatus, mitochondria, endosomes, plasma membrane, and vacuolar membrane (17). In yeast, septin mutations result in defective autophagy. Specifically, the retrograde transport of Atg9 is impaired, suggesting that septins may assist Atg9 vesicles in supplying membranes for the phagophore (122, 123). In line with this mechanism, under autophagy-inducing conditions, septins localize to the cytoplasm and assemble into non-canonical septin rings at the PAS (17, 122), and physically interact with the autophagy proteins Atg8 and Atg9 (122, 124). Furthermore, in mutants deficient in early autophagosome formation (such as atg1Δ), septin localization at the PAS decreases (122). The above evidence suggests that septins play a crucial role in the early stages of autophagy in yeast (122).

In recent years, studies have also revealed that septins are involved in autophagy of intracellular bacteria induced by bacterial infection in mammalian cells (125, 126). Upon bacterial infection, septins assemble into cage-like structures around intracellular bacteria, thereby restricting bacterial motility and recruiting autophagic markers to target them for autophagy-mediated degradation (127–129). Furthermore, septins can function in membrane remodeling and can associate directly with membranes in a curvature-dependent manner, regulating vesicle fusion (130–132).

Septins crosslink with actin or microtubules to spatially restrict inflammasome assembly. The Septin complex can bind NLRP3, preventing its nonspecific activation, while dissociating upon pathogen invasion to initiate inflammasome assembly (133). Septins regulate membrane domain partitioning in dendritic cells (DCs), confining MHC II molecules to the immunological synapse region, thereby enhancing antigen presentation efficiency. Septins form ring−like structures around cytokine−containing vesicles, regulating the efficiency of vesicle–membrane fusion. Septins are also involved in regulating vesicle trafficking during endocytosis and exocytosis, including the transport of internalized cargo and lysosome delivery (134). In neurons, Septins modulate local actin dynamics to influence dendritic spine (post−synaptic structure) plasticity (135).

In summary, as a conserved family of eukaryotic cytoskeletal proteins, septins play fundamental roles in basic cellular processes such as cell division, morphogenesis, and membrane trafficking by forming diffusion barriers and molecular scaffolds. More recently, septins have been recognized as important coordinators within the autophagy regulatory network, contributing to both pathogen clearance and basal autophagy. Nevertheless, critical questions remain regarding the mechanisms of septin function in conventional autophagy in mammals, the regulatory signals governing their dynamic subcellular localization, and the fine-tuning of their autophagic roles by post-translational modifications.

## 6 Cytoskeleton–autophagy regulation in immune cells

Autophagy is an essential intracellular quality control system that selectively eliminates inflammation-driving factors, such as reactive oxygen species (ROS), damage-associated molecular patterns (DAMPs), and damaged cells. This process negatively regulates the excessive activation of inflammasomes like NLRP3, thereby mitigating inflammatory responses (136). Meanwhile, the dynamic remodeling of the cytoskeleton, particularly microtubules and microfilaments, provides the structural basis for inflammatory signal transduction and execution (83). Specifically, actin participates in the assembly and subcellular localization of inflammasomes and mediates the activation of caspase-1 (137). Microtubules, serving as the primary intracellular transport tracks, facilitate the non-classical secretion of signal peptide-lacking inflammatory cytokines such as IL-1β and IL-18 (138, 139).

In neuroimmune responses, cytoskeletal regulation is critical for immune cell function. The microtubule and actin cytoskeletons provide structural support for the platform of inflammasome assembly (83), whereas autophagy can suppress excessive inflammasome activation by clearing damaged mitochondria and other stimuli (140). Consequently, impaired autophagy leads to exacerbated inflammatory responses. The Arp2/3 complex serves as a critical node in initiating this process; its inhibition not only disrupts the formation of the phagocytic cup (141), but also impairs specialized forms of autophagy such as LC3-associated phagocytosis (40). This results in the accumulation of toxic materials, including Aβ (141), and is accompanied by sustained NLRP3 inflammasome activation and maturation of IL-1β (142).

In inflammation regulation, the cytoskeleton supports the initiation, transduction, and effector release of inflammatory signals through structural and transport mechanisms (83, 138, 143). In contrast, autophagy exerts terminal inhibitory effects by clearing inflammatory triggers and overactivated signaling components (23, 140). Their functions are coupled to maintain a dynamic balance. Disruption of this synergistic mechanism—such as impaired autophagy accompanied by abnormal activation or altered stability of the cytoskeleton—leads to persistent activation of inflammasome signaling (136), excessive release of mature inflammatory factors, and ultimately the progression of chronic and pathological inflammation (144). This mechanism holds significant implications for neuroinflammation, autoimmune diseases, and age-related disorders.

As the resident immune cells of the central nervous system (CNS), microglia exert pivotal roles in maintaining homeostasis and orchestrating responses to infection (145). Its anti-infection immune function is highly dependent on the dynamic reorganization of the cytoskeleton (146). Meanwhile, autophagy plays a key role in restraining metabolic and signaling pathways (147). The cytoskeleton empowers microglia to phagocytose apoptotic neurons and pathogenic protein aggregates like Aβ, a hallmark of Alzheimer’s disease, with autophagy subsequently degrading these phagocytosed cargoes. This clearance mechanism initiates when receptors such as Triggering Receptor Expressed on Myeloid Cells 2(TREM2) (148) and CR3 (149) recognize opsonins like C1q and C3b on pathological substrates (150). This triggers cytoskeletal remodeling, enabling the extension of pseudopods for engulfment and the formation of a phagosome, which matures into a degradative phagolysosome upon fusion with lysosomes (151). Cytoskeletal dysfunction can disrupt this critical engulfment process and impair autophagic clearance, leading to the accumulation of neurotoxic materials, a key event in neurodegeneration. Furthermore, microglia employ F-actin-based tunneling nanotubes (TNTs) to connect with distressed neurons. Through TNTs, microglia not only alleviate neuronal proteotoxicity by importing and degrading misfolded proteins but also provide metabolic support via the transfer of healthy mitochondria. This exchange helps restore neuronal bioenergetics, mitigate oxidative stress, and ultimately promote neuronal survival in the context of neurodegenerative pathology (152). Dynamic actin reorganization serves as the structural basis for the chemotactic migration (153) and phagocytic clearance functions of microglia (154). Autophagy provides metabolic support by clearing damaged organelles, particularly mitochondria, promoting the maintenance of an anti-inflammatory/repair phenotype in microglia (155).

Both microglia and infiltrating macrophages can be classified into pro-inflammatory M1-like and anti-inflammatory/repair M2-like phenotypes based on their functional states (156, 157).M1-like cells primarily rely on glycolysis as their main metabolic pathway (155, 158, 159), and their autophagy levels are typically suppressed. In contrast, M2-like cells predominantly depend on oxidative phosphorylation (158, 159). Autophagy in these cells supports the anti-inflammatory M2 phenotype by degrading inflammasomes (such as NLRP3) and inflammatory signaling molecules (23), thereby providing metabolic support and suppressing pro-inflammatory M1-like responses (155). The cytoskeleton, serving as the structural basis for autophagosome formation and transport, plays a key role in this regulation (160). If cytoskeletal reorganization is disrupted, leading to impaired autophagic flux (161), these cells tend to polarize toward the pro-inflammatory M1-like phenotype, releasing large amounts of cytokines such as IL-1β and TNF-α, which exacerbate neuroinflammation (155).

Additionally, similar to microglia, infiltrating macrophages rely on actin-driven phagocytic cup formation to clear apoptotic cells and myelin debris (162). Subsequently, the phagosomes need to fuse with autophagosomes/lysosomes to complete degradation (163). During migration, infiltrating macrophages also depend on the rapid reorganization of the actin cytoskeleton to form pseudopods, thereby facilitating movement toward inflammatory sites (164). Autophagy not only provides energy and biosynthetic precursors for these highly energy-demanding processes (161), but its own transport also relies on the cytoskeleton (160).

Astrocytes play crucial roles in maintaining the blood-brain barrier and supporting neuronal function (165), while also exhibiting immune functions, particularly during central nervous system (CNS) injury or inflammation (166). Autophagy plays a significant role in the metabolic regulation and immune responses of astrocytes (167). The actin cytoskeleton regulates the movement of autophagic vesicles in astrocytes, aiding in the clearance of toxic substances or damaged organelles (36). During CNS inflammation, astrocytes undergo alterations in cytoskeletal structure (168),which affect core functions such as the clearance of toxic proteins (169), maintenance of mitochondrial homeostasis (170), and regulation of neuroinflammation (171)by modulating the transport of autophagic vesicles and their fusion with lysosomes. This, in turn, either promotes or suppresses the transmission of inflammatory signals (172). Dysregulation of this “cytoskeleton-autophagy” axis is a critical factor leading to astrocyte dysfunction and exacerbating disease progression.

During T cell activation, the cytoskeleton undergoes reorganization and forms an immune synapse (112). This structure not only serves as the center for T cell receptor (TCR) signal transduction (143) but also, through its downstream signaling, suppresses autophagy (173). This promotes a metabolic shift toward glycolysis and facilitates the differentiation of effector T cells (174). If cytoskeletal function is impaired, it may lead to dysregulated autophagy, disrupt metabolic reprogramming, and ultimately weaken the responsiveness of effector T cells. Unlike short-lived effector T cells, memory T cells, which serve as the cornerstone of long-term immune protection, primarily rely on oxidative phosphorylation and fatty acid oxidation for energy (175). Autophagy is crucial for maintaining their metabolic adaptability and long-term survival (176).

In antigen-presenting cells, autophagy participates in processing intracellular antigens and loading them onto MHC class II molecules, thereby initiating T cell adaptive immune responses (177). The fusion of autophagosomes with MHC class II loading compartments depends on microtubule transport (177). Disruption of cytoskeletal function may reduce antigen presentation efficiency and weaken T cell immune responses. Similarly, T cell migration to sites of infection or tumors heavily relies on the rapid polymerization and depolymerization of the actin cytoskeleton to form pseudopods and alter cell morphology (178). Autophagy also provides energy and raw materials for this process (161) and can dynamically regulate cytoskeletal plasticity by degrading its components, thereby precisely controlling T cell motility (179).

The Arp2/3 complex regulates T cell homeostasis by maintaining TCR endosomal trafficking and surface expression. Specific knockout of Arpc2 in T cells leads to reduced numbers of peripheral T cells and impaired homeostasis (180). WASP serves as a key activator of the Arp2/3 complex. Its deficiency impairs the effective nucleation of Arp2/3, leading to compromised immunological synapse formation, weakened TCR signaling, reduced T cell numbers, and functional abnormalities (181). The Arp2/3 complex regulates the migration and force generation of dendritic cells, affecting their movement efficiency toward the T−cell zone in lymph nodes. This impairment can weaken antigen presentation and T−cell activation capacity, thereby compromising the initiation and coordination of neuroimmune responses (182). Knockout of the Arp2/3 complex also impairs integrin-dependent phagocytosis, adhesion, and haptotactic migration in macrophages, while preserving or even enhancing certain chemotactic migration capabilities (183). The Arp2/3 complex is essential for the formation of neutrophil extracellular traps (NETs).Inhibiting its function blocks the nuclear translocation of neutrophil elastase (NE), thereby suppressing NETosis and affecting anti−infective immunity, among other processes (184).

## 7 Cytoskeleton–autophagy axis in neuroimmune disorders

The cytoskeleton-autophagy axis collectively determines the direction of central nervous system immune responses—toward homeostatic repair or destructive chronic inflammation—by regulating microglial phenotype switching, glial cell reactivity, BBB integrity, and peripheral immune cell infiltration. Dysfunction of this regulatory axis is a core mechanism underlying the onset and progression of neuroimmune disorders. Although clinical evidence is still limited, a number of animal and *in vitro* studies have yielded supportive findings.

In Drosophila brain aging, accumulation of F-actin leads to the buildup of autophagy markers Atg8a and p62, disrupting autophagic flux (185). Reduction of F-actin can restore autophagic activity and decrease the aggregation of ubiquitinated proteins (186). Excessive F-actin also inhibits mitophagy, resulting in the accumulation of damaged mitochondria; reducing F-actin accumulation restores autophagy, clears protein aggregates, promotes mitochondrial degradation, delays brain aging, and extends lifespan (185). In chronic neurodegenerative diseases, autophagy dysfunction interrupts metabolic support (187),impairing the capacity for actin reorganization. Consequently, microglial migration and phagocytic efficiency decline, while polarization toward a pro-inflammatory phenotype increases, forming a vicious cycle of pathological protein accumulation and persistent neuroinflammation (188).

The integrity of the blood-brain barrier (BBB) depends on the homeostasis of endothelial cells and the dynamic balance between autophagy and the cytoskeletal network (189). The cytoskeleton forms the physical foundation of the BBB (189) and is directly coupled with tight junction (TJ) proteins, constituting the structural basis of the barrier (190). Under physiological conditions, autophagy maintains the stability and function of TJ proteins by clearing damaged mitochondria and protein aggregates, thereby suppressing oxidative stress and ensuring proper synthesis and assembly of tight junctions. Under stress conditions such as neuroinflammation or ischemia, impaired autophagy leads to intracellular damage accumulation, which abnormally activates pathways like Rho/ROCK. This triggers pathological actin reorganization (e.g., stress fiber formation), directly disrupting the anchoring and connectivity of TJ proteins, ultimately resulting in a sharp increase in BBB permeability and functional breakdown (191).

There is growing preclinical evidence that modulating microtubule stability and tubulin post-translational modifications can influence neuroimmune phenotypes. Microtubule-stabilizing agents (e.g., epothilones) and inhibitors of tubulin deacetylases (e.g., HDAC6 inhibitors) restore axonal transport, reduce pathological protein accumulation, and attenuate glial activation in several neurodegenerative models, although clinical translation remains limited by blood–brain barrier permeability and toxicity concerns.

Microtubule destabilizers (e.g., Colchicine, Vincristine, Podofilox) function by inhibiting microtubule polymerization, which impedes autophagosome transport and may initially result in autophagosome accumulation (192); however, prolonged exposure has been shown to activate endoplasmic reticulum stress and induce protective autophagy (193).Furthermore, microtubule destabilizers disperse the Golgi apparatus, delay the degradation of STING protein, and enhance interferon signaling, thereby strengthening anti-tumor immune responses (194). The consequent release of ATP and other molecules can further activate the NLRP3 inflammasome in tumor-associated macrophages, fostering a pro-inflammatory microenvironment (195). In contrast, microtubule stabilizers (e.g., Paclitaxel, Docetaxel) can enhance the efficiency of autophagosome-lysosome transport (192), however, high doses may trigger excessive autophagy, leading to cytotoxicity (196) (197). Beyond their direct cytotoxic effects, these agents promote dendritic cell maturation, regulate macrophage polarization (198), and exhibit synergistic effects when combined with immune checkpoint inhibitors (199).

Tubastatin A, a specific HDAC6 inhibitor, upregulates Hsc70 and Lamp2A expression, activates chaperone-mediated autophagy, and promotes the degradation of α-synuclein. Additionally, it reduces Ser129 phosphorylation of α-synuclein, mitigates astrocyte reactivity, and alleviates neuroinflammation, thereby protecting dopaminergic neurons in the substantia nigra of rats. These findings underscore the therapeutic potential of Tubastatin A in synucleinopathies, including Parkinson’s disease (200). Tubastatin A, another HDAC6 inhibitor, can restore the acetylation level of α-tubulin in ischemic brain regions and improve the transport of autophagic-related organelles such as mitochondria. Meanwhile, it upregulates fibroblast growth factor-21 (FGF-21) and its downstream signaling pathways, including Extracellular Signal-Regulated Kinase(ERK) and Akt/Glycogen Synthase Kinase 3β(GSK-3β). In both the rat middle cerebral artery occlusion model and the cortical neuronal excitotoxicity model, Tubastatin A significantly reduces cerebral infarct area, protects neurons from excitotoxic damage, and extends the therapeutic time window to 24 hours post-ischemia, demonstrating notable neuroprotective effects (201). Epothilone B enhances microtubule stability by promoting αTAT1-dependent acetylation of α-tubulin, thereby preserving cytoskeletal integrity and improving axonal transport. This is reflected in the upregulation of motor proteins kinesin and dynein, which provides a critical structural foundation for the directional transport of autophagy-related cargo. In a mouse model of intracerebral hemorrhage, the compound significantly reduced the loss of dopaminergic neurons in the substantia nigra, promoted the repair of the nigrostriatal pathway, and improved fine motor function. Importantly, no significant side effects were observed at effective doses. Together, these findings indicate that the neuroprotective effect of Epothilone B is achieved primarily through the maintenance of microtubule stability (202).

MLi2 and PF-06447475, as inhibitors of Leucine-Rich Repeat Kinase 2(LRRK2) kinase activity, demonstrate significant effects in mouse models of Alzheimer’s disease and Parkinson’s disease. They reduce glial cell activation and the release of pro-inflammatory factors, while enhancing autophagic flux to effectively clear abnormal proteins and interrupt the vicious cycle of neuronal damage. Additionally, these compounds suppress cytotoxic responses, indicating their therapeutic potential for a range of neurodegenerative diseases (203). GSK3357679A, an LRRK2 inhibitor, mitigates mitophagy defects in G2019S mutant mice by activating the ATG5-dependent canonical autophagy pathway, independently of the PINK1/Parkin axis. This protective effect on dopaminergic neurons supports its potential as a therapeutic strategy for Parkinson’s disease (PD) (204).

Fasudil and Y27632, representative ROCK inhibitors, promote autophagy in HMC3 cells (as evidenced by an increased LC3b-II/LC3b-I ratio), reduce α-synuclein aggregation, and inhibit its internalization by microglia. Furthermore, these inhibitors attenuate oxidative stress, suppress intracellular calcium elevation, and mitigate neuroinflammation, collectively contributing to the protection of dopaminergic neurons and highlighting their therapeutic potential for Parkinson’s disease (205). ROCK inhibitors such as SR3677 and Y27632 enhance Parkin-mediated mitophagy by inhibiting ROCK2, promoting HK2 phosphorylation and its subsequent recruitment to mitochondria, thereby accelerating the degradation of damaged mitochondria. In both cellular and Drosophila models, these interventions improve mitochondrial function, increase cell viability, extend lifespan, restore motor capacity, and ultimately exert neuroprotective effects (206). Fasudil enhances autophagic activity, as evidenced by an increased LC3-II/LC3-I ratio, and promotes α-synuclein degradation through the inhibition of ROCK2 activity, which activates the c-Jun N-Terminal Kinase 1(JNK1)/B-Cell Lymphoma 2(Bcl-20/Beclin-1 and Akt/mTOR pathways. In an AAV-mediated rat model of Parkinson’s disease, this treatment significantly reduced the loss of dopaminergic neurons in the substantia nigra, improved striatal dopaminergic function, and alleviated motor behavioral deficits. Furthermore, Fasudil has demonstrated anti-inflammatory potential. These results suggest that Fasudil holds translational promise as a disease-modifying therapeutic strategy for Parkinson’s disease (207).

Actin polymerization inhibitors (e.g., Cytochalasin D, Latrunculin A), by inhibiting actin polymerization, these compounds hinder the extension of autophagic precursor membranes and significantly reduce autophagosome formation (36). Immunologically, they disrupt the pseudopod structure of macrophages, thereby impairing phagocytic capacity (208). Vimentin modulators affect the extracellular matrix (ECM) degradation capacity of macrophages (209).

## 8 Conclusion and perspectives

This article systematically elucidates the regulatory network between the cytoskeleton—including microfilaments, microtubules, intermediate filaments, and the Septin family—and autophagy (**Table 1**). The cytoskeleton not only provides essential structural support and transport tracks for the autophagic process but also serves as a dynamic signaling integration platform, actively participating in every stage of autophagy, including initiation, nucleation, elongation, transport, and fusion. Microfilaments play a central role in autophagosome formation and selective autophagy through dynamic polymerization and depolymerization, Arp2/3 complex-mediated nucleation, and “physical collecting” mechanisms. Microtubules, via dynamic instability, the “tubulin code,” and motor proteins, regulate autophagic signaling, directional transport of autophagosomes, and their fusion with lysosomes. Intermediate filaments (such as vimentin) and septins influence the autophagic flux by controlling organelle localization, forming diffusion barriers, and recruiting autophagy-related proteins.

**Table 1:** Summary of the functions and regulatory mechanisms of various components of the cytoskeleton in autophagy and Neuroimmune functions.

Cytoskeleton system	Function in autophagy	Key role process	Representative regulatory molecules/structures	Association with disease/function	Neuroimmune Relevance & Implications	
Actin	Autophagosome biogenesis, mechanical stress perception and conduction, selective autophagy substrate “collection”, autophagy flow regulation	Provide mechanical support and driving force;Arp2/3-mediated nucleation; Aggregation/depolymerization dynamics; The act of riding a bicycle; Collaborate with molecular motors	Arp2/3 complex、JMY、Myo1D、mTORC1、RhoA/ROCK、Nesfatin-1、ADF7	Brain aging (210),cancer cell proliferation and metastasis (211)	Regulates microglial phagocytosis, immune synapse formation, and cytokine release; Dysfunction linked to neuroinflammation.	
Microtubules	The “orbit” of autophagosome transport, autophagy initiation signaling platform, autophagosome lysosome fusion, organelle localization	Dynamic instability; Microtubule code “(acetylation, etc.);Motor protein (motor protein, motor protein) transport;MTORC1 signal regulation	HDAC6、Sirtuin 2, (SIRT2)、PLK4、TFEB/TFE3、ULK1、Beclin 1	Cancer (212), ciliary development (97),meiosis (75),embryonic development (213)		Critical for immune cell (e.g., T cell, microglia) migration, polarity, and lysosome positioning; Targeted in neuroinflammatory diseases.
Intermediate Filaments	Maintain normal distribution of autophagosomes, promote autophagosome lysosome fusion, regulate mTORC1 activity, and anchor organelles	Form a network backbone; As a diffusion barrier; Regulating the spatial localization of organelles; Affects signaling pathways (such as mTOR)	Vimentin, Keratin, Withaferin A (WFA)	Cancer (214), hepatocyte autophagy (97) neurodegenerative diseases (215)	Vimentin stabilizes NLRP3 inflammasome in macrophages; GFAP affects microglial-synaptic contact; Key for immune cell structural integrity.	
Septin	The spatiotemporal coordinator of autophagy, serving as a scaffold for bacterial autophagy, and involved in the early stages of autophagy in yeast (PAS assembly, Atg9 vesicle transport)	Forming diffusion barriers and molecular scaffolds; Associate membrane structures in a curvature dependent manner; Recruiting autophagy related proteins; Interaction with Atg8/Atg9	CDC3/10/11/12 (yeast), SEPTIN2/7/9 (mammals) Atg8/LC3、Atg9	Bacterial infection (125), neurodegenerative diseases (216), cancer metastasis (217)	Forms cages around cytosolic pathogens for autophagy (xenophagy); May regulate inflammasome assembly and neuronal spine plasticity.	

In the future, it will be important to elucidate the complex crosstalk and signaling integration among the microtubule, microfilament, and intermediate filament systems, and to clarify how these interactions coordinate and guide the complete dynamics of autophagic flux. Also, it is necessary to precisely dissect the specific regulatory patterns of the cytoskeleton–autophagy axis under distinct physiological and pathological contexts—such as cellular senescence (aging) or neurodegeneration. Ultimately, understanding the interplay between the cytoskeleton and autophagy from the perspectives of mechanobiology and dynamic membrane biology will provide deeper insights into the maintenance of cellular homeostasis. Based on the mutual regulatory network between the cytoskeleton and autophagy, targeting the cytoskeleton–autophagy axis holds promise for developing novel therapeutic strategies for a variety neuroimmune diseases, including neurodegenerative disorders.
